# Safe Sleep Knowledge and Use of Provided Cribs in a Crib Delivery Program

**Published:** 2017-08-30

**Authors:** Matthew Engel, Carolyn R. Ahlers-Schmidt, Bonita Suter

**Affiliations:** 1 University of Kansas School of Medicine-Wichita, Department of Pediatrics; 2 Van Buren Intermediate School District, Lawrence, MI

**Keywords:** infant mortality, sudden infant death, infant equipment, health education

## Abstract

**Background:**

Risk of infant sleep-related death can be reduced through safe sleep practices. Barriers to infant safe sleep have been mitigated through education and crib distribution, however, previous studies have not explored whether distributed cribs are put to use.

**Methods:**

In a rural Michigan county, the Great Start Sleep Initiative supplied cribs and education shortly after infant birth to families with high-risk of infant mortality, as assessed through comprehensive interviews with families by program staff. Participant knowledge was evaluated using structured pre- and post-assessments before and after education. Further, a home visit was conducted to evaluate the infant’s sleeping environment. Data from the program, collected between January 2012 and December 2014, was evaluated.

**Results:**

Cribs and concomitant education were delivered to 75 caregivers. Knowledge of safe sleep practices increased significantly at follow-up with 67 caregivers (89%) affirming back positioning, 68 (91%) endorsing removal of unsafe items or soft objects, such as blankets, from the sleeping area, and 42 (56%) renouncing bed-sharing. At the home visit, 74 caregivers (99%) were using a crib to put their infant down to sleep, 70 (93%) were using the provided crib, and 67 (89%) had no unsafe items in the child’s sleeping area.

**Conclusions:**

Providing education to high-risk mothers resulted in improved safe sleep knowledge and provided cribs are used in these homes.

## Introduction

Infant mortality is a critical measure of population health. Despite declines following the 1994 launch of the Back-to-Sleep campaign, sudden infant death syndrome (SIDS) remains a leading cause of infant death.^1^,^2^ Although the number of SIDS diagnoses decreased overall secondary to supine sleep position recommendations, other types of sleep-related infant deaths, including accidental suffocation and strangulation, increased.^3^ This led the American Academy of Pediatrics to revise its guidelines to emphasize sleep environment as well as positioning.^4^,^5^

In August of 1998, the Cribs for Kids campaign was launched in Allegheny County, Pennsylvania.^6^ This program distributed full-sized cribs and mattresses, along with written educational materials, to low-income families. Following this initiative, the local SIDs rate dropped by 63%. Parents reported that without the Cribs for Kids program, babies would have slept in an adult bed, on the floor, or other unsafe location. The Cribs for Kids program became a model for resource provision and behavior change among low-income individuals and similar programs have been adopted in many states.

In Michigan, about 120 infants die suddenly and unexpectedly in unsafe sleep environments each year.^7^ In Van Buren County, the need for further safe sleep education and resources was identified through home visiting programs and child death reviews. The Great Start Safe Sleep Initiative was created to provide one-on-one training on safe sleep practices for families with demonstrated need and a safe sleep set containing a portable crib, an infant wearable blanket, two fitted sheets, and literature on safe sleep. Need was determined holistically by maternal infant health program (MIHP) staff, with indicators including low-income, racial minorities, and migrant worker status. The staff ultimately make the determination of need after meeting and interviewing the family. The majority of sleep sets were delivered by MIHP staff who were involved with these families. The MIHP also conducted follow-up visits, giving staff an opportunity to evaluate the impact of safe sleep training.

Prior to delivery of the safe sleep set, a survey was conducted with a caregiver from each family to determine knowledge about safe sleep. Approximately two months after the sleep set was delivered, the safe sleep trainer contacted the caregiver by telephone to discuss any concerns and administer a survey to check knowledge retention. The trainer reminded the caregiver of items they have forgotten and scheduled a follow-up home visit. At the visit, the trainer completed a checklist by interviewing caregivers and viewing the portable crib to ensure that only one child was using the crib, that the crib was assembled properly, no hazards were nearby, and that no soft objects were in the crib. The results were reviewed with the parent and any concerns are addressed.

The purpose of this study was to analyze home visit reports and changes in knowledge from this program.

## Methods

The Great Start Sleep Initiative provided de-identified data for all mothers with a follow-up home visit between January 2012 and December 2014. These data were collected originally for program purposes and contained caregiver responses to the two knowledge questionnaires and the checklist completed by the MIHP staff. This study was determined to be exempt from review by the University of Kansas School of Medicine-Wichita Human Subjects Committee.

Caregivers were asked on both surveys what they knew about safe sleep; open-ended responses were summarized post-hoc across four themes (back positioning, crib/bassinet location, no bed sharing, and no items in the crib) and coded as safe/unsafe. Knowledge was compared between both caregiver questionnaires using McNemar’s tests. The home visitor’s checklist was summarized to determine how many parents had a crib assembled and in use at the time of visit; written comments by MIHP staff were reviewed. Analysis was conducted in June 2015.

## Results

In total, 75 families were included in this evaluation. Infants were predominantly Hispanic (61%) with an average age of seven weeks (SD = 12) at referral. On average, cribs were delivered when the child was nine weeks old (SD = 12), and home visits were completed at 22 weeks (SD = 12). Put another way, cribs were delivered two weeks (SD = 4) after referral and home visits were conducted 13 weeks (SD = 6) after crib delivery.

Reported household size ranged from two to 11 individuals with a median of five. On the pre-test, 62 respondents (85%) indicated they had received safe sleep education previously. Only four respondents indicated a friend or family member had given them information about safe sleep. Other major sources of safe sleep information were community services (n = 40, 53%) and health care providers (n = 36, 48%), including doctors, nurses, and hospital workers.

Respondents’ knowledge was measured across the four identified themes. At baseline, 44 respondents (59%) were aware of back positioning, 40 (53%) noted that a crib was part of a safe sleep environment, 53 (71%) mentioned eliminating unsafe items from the sleep environment, and 23 (31%) explicitly addressed not bed sharing (Table 1). At follow-up, knowledge of back positioning, removing items from sleeping area, and avoiding bed sharing improved. While a higher proportion of respondents noted the importance of a crib as a safe sleep environment, the difference was not statistically significant.

Regarding intentions at time of referral, 30 respondents (48%) indicated that they would place their infant in a crib, portable crib, or bassinet to sleep, 36 (59%) reported they would put nothing other than a fitted sheet in the place where their baby sleeps, 66 (92%) reported not allowing smoking indoors, and 56 (85%) affirmed that if they smoked they change their clothes before holding their baby (Table 1). Reports of safe location and eliminating hazards in the sleeping area were improved following crib delivery. Smoking reports were not significantly different on follow-up.

At the home visit, 68 households (91%) had the provided crib present and assembled. An additional two households (3%) had the crib travel with the child to daycare, explaining its absence at the time of the visit. Among households without the provided crib, two received a separate full-sized crib that they were using while keeping the portable crib at another caregiver’s house, one had damaged the crib irreparably and disposed of it, and one said their infant disliked sleeping in the crib. In total, 73 households (97%) had a crib set up for their infant to sleep in. Three households (4%) had clothes or blankets draped over the edge of the crib, but no other unsafe sleep elements were observed in cribs by the home visitor.

## Discussion

Previous studies have shown that caregivers can be knowledgeable about infant safe sleep but do not always follow recommendations.^8^,^9^ To the authors’ knowledge, this was the first study to show that, when provided cribs, most caregivers use them and maintain a safe sleep environment for their infant. Providing concomitant cribs and education to mothers resulted in improved safe sleep knowledge and use of the crib months after these resources were provided. Few mothers obtained other cribs, and only one infant was reported not to sleep in a crib.

One study limitation is that the families followed in this study are potentially non-generalizable, coming from one Midwestern county. Also, while mothers may not have otherwise found a way to obtain a crib, it was determined prior to crib delivery that families could not afford a crib and very few mothers obtained another crib from a friend or family member. Finally, while home visitors were able to assess the sleep environment, visits were in the middle of the day and no observations were made of infants asleep in the cribs. Despite these limitations, given that no previous studies have evaluated the at-home use of cribs from similar distribution programs, this evidence supported future growth and exploration of these programs. Studies should continue to evaluate whether these findings are replicable in other communities.

## Conclusion

For at-risk families, the lack of resources, namely a crib, may be the primary reason for putting infants in unsafe sleep environments. In this sample, cribs distributed to families in need were employed, effectively reducing the risk of sleep-related death for these infants.

## Figures and Tables

**Table 1:** Comparison of pre- and post- surveys. Data were collected between January 2012 and December 2014.

	Pre n=75*	Post n=75**	p-value
**Knowledge, n (%)**			
Back positioning	44 (59)	67 (89)	<0.001
In a crib or bassinet	40 (53)	33 (65)	0.078
No items in sleep area	53 (71)	68 (91)	0.003
No bed sharing	23 (31)	42 (56)	0.003
**Intentions/Behaviors, n (%)**			
Safe Location	30 (48)	74 (99)	<0.001
No items in sleep area	36 (59)	67 (89)	0.002
No smoking indoors	66 (92)	71 (95)	0.500
Avoid third hand smoke exposure	56 (85)	62 (84)	0..454

* n for *No smoking indoors* = 72, *Safe Location* = 62, *No items in sleep area* = 61, *Avoid third hand smoke exposure* = 66.

** n for *In a crib or bassinet* = 51, *Avoid third hand smoke exposure* = 74
